# The relationship between niche breadth and phylogenetic characteristics of eight species of rhubarb on the Qinghai‐Tibet Plateau, Asia

**DOI:** 10.1002/ece3.11040

**Published:** 2024-02-29

**Authors:** Kaiyang Chen, Bo Wang, Chen Chen, Guoying Zhou

**Affiliations:** ^1^ Northwest Institute of Plateau Biology, Chinese Academy of Science Xining China; ^2^ University of Chinese Academy of Sciences Beijing China

**Keywords:** niche breadth, niche overlap, phylogenetic, range overlap, rhubarb

## Abstract

The relationship between spatial distribution and phylogeny has been widely debated in recent decades. To understand biogeographic and evolutionary history relationships and to explore the interspecific similarities and phylogenetic correlations of niche characteristics, we collected and recorded all distribution points for eight species of rhubarb on the Qinghai‐Tibet Plateau, used different methods to describe the ecological niche, and explored the relationship between phylogeny, ecological niche, and distribution range. The results reveal that: (1) the ranges of optimal environmental variables for species with close kinship are not exactly the same, ecologically similar species are not necessarily sister species, and the overlap of rhubarb has no significant correlation with phylogeny. Therefore, the impact of ecological dimensions on species formation is greater than that of geographical latitude for the eight species of rhubarb. (2) Among the eight species of rhubarb, the breadth of ecological niche is positively correlated with the current suitable habitat area and negatively correlated with fluctuations in future suitable habitat area. In the future, except for *Rheum tanguticum* and *Rheum palmatum*, the suitable planting areas for the other six species of rhubarb will decrease as greenhouse gas emissions concentrations and time increase. Therefore, species with smaller ecological niches are at a greater risk of habitat loss compared to species with larger ecological niches. (3) In both existing and future distribution prediction models of rhubarb, we observed that both the widely distributed *Rheum spiciforme* and the niche narrow *Rheum nobile*, all eight species of rhubarb are present in the Hengduan Mountains, based on our analysis, we propose that the Hengduan Mountains should be regarded as a priority conservation area for rhubarb, to preserve the species' biodiversity. Our study lays the groundwork for identifying evolutionary trends in ecological specialization.

## Graphical abstract

The current and future habitat range of eight species of Rhubarb distributed on the Tibetan Plateau and the environmental factors affecting their distribution. The relationship between niche and kinship.

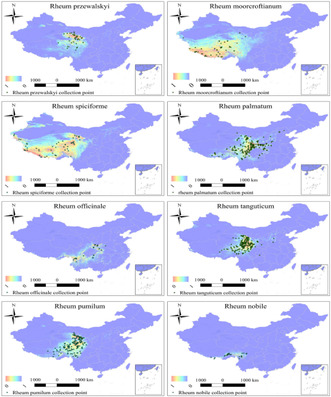

## 1 INTRODUCTION

The correlation between spatial distribution and phylogeny has received extensive attention in recent decades (Savolainen et al., 2013). Geographic isolation is a prerequisite of reproductive isolation. Kneitel (2019) demonstrated that for any species in any region, the closest related species are unlikely to be present in the same area or remote areas and will instead exist in adjacent areas separated by some type of barrier (Losos et al., 2003). In stable environments, niche overlap between closely related species may generate habitat competition to a certain extent (Baldwin, 2005), considering that two species occupy the same ecological niche (Baldwin, 2005; Runquist & Stanton, 2012). It should be noted that resource‐limited and species with the same niche and resource use patterns cannot coexist for a long period (Anacker & Strauss, 2014; Noden, 1986; Penny, 2004). However, phylogenetic niche conservation (PNC) considers the tendency of phylogenetically close species to maintain their ecological similarity during evolution. Fundamentally, PNC refers to the lack of adaptability of a species to new environmental conditions at a given time scale, and close relatives are more likely to gather in the same area (Harvey & Pagel, 1991). Therefore, the relationship between phylogeny and spatial distribution has not been clearly elucidated in recent decades.

The range of a plant is often defined as the two‐dimensional extent of the spatial distribution of a species based on latitude and longitude ranges (Gaston, 1991), and there are a variety of factors that affect the geographic distribution of plants. First, the geographic distribution of plants is undoubtedly sensitive to environmental factors (Louthan et al., 2015; Sosef et al., 2017) that directly affect community composition and population dynamics (Gao et al., 2019). For example, *Sinadoxa corydalifolia* is an alpine plant, and temperature seriously affects its reproductive success, so *Sinadoxa corydalifolia* is only distributed in low‐temperature areas (Huang et al., 2020); *Meconopsis* is a plateau plant, and the elevation determines plant survival range. Additionally, *Meconopsis* does not grow in plains and low‐elevation areas (He et al., 2019; Wang et al., 2021). Second, niche breadth has been confirmed to have a significant relationship with distribution range according to previous studies (Cai et al., 2021; Slatyer et al., 2013; Zelen & Chytr, 2019). Species with broad niches have more areas available to them as suitable habitats (Brown, 1984; Sheth et al., 2013) and possess correspondingly larger potential geographic ranges (Boulangeat et al., 2012; Stephan et al., 2018). Taking the North American tree line as an example, these tree species are more able to tolerate greater variability in local climatic condition and, adapt to harsh soil conditions and weaker environmental energy levels, and exhibit a larger growth range (Morin & Lechowicz, 2013). Third, range change and migration of plants are important strategies for coping with climate change (Parmesan & Yohe, 2003). Numerous studies have indicated that plants respond to future climate change by shifting their distribution to higher latitudes and/or elevations (Baker & Moseley, 2007; Pauli et al., 2010; Rumpf et al., 2018) such as the advancement of the Himalayan‐Hengduan shrub line to higher elevations in recent decades (Thuiller et al., 2005). Therefore, determining the species niche breadth can help identify species for priority conservation actions (Savolainen et al., 2013), and predicting different future patterns of atmospheric development can estimate the spatial distribution and extent of the suitable habitat of a species.

The Qinghai‐Tibet Plateau is considered the third pole of the world (Wang et al., 2006; Ye, 1981), and many endemic species are distributed throughout this area (Madriñán et al., 2013; Xin‐Hui et al., 2014; Yang et al., 2014). During the Quaternary period, global temperature dropped sharply (He et al., 2019). The height of the Qinghai‐Tibet Plateau generally increased (Su, Farnsworth, et al., 2019; Wang et al., 2021). Species in shared environments tend to evolve similar adaptations under the influence of a given phylogenetic background (Gaston & Blackburn, 1997; Kramer et al., 2010) with species rapidly forming to adapt to changing conditions (Godwin, 1943; Jaime et al., 2015; Qiao et al., 2016). Certain researchers believe that most temperate plants originate primarily from the Qinghai‐Tibet Plateau and its adjacent plateaus (Godwin, 1943; Jia et al., 2012; Rumpf et al., 2018; Wu, 1996). Rhubarb is a perennial herb of the *Polygonaceae* family, and there are approximately 60 species worldwide that are primarily distributed in temperate and subtropical alpine mountains in Asia. Rhubarb has a history of thousands of years in China (Zheng et al., 2021), it has the effects of regulating the stomach, anti‐tumor, anti‐inflammatory, liver protection, and delaying aging. (Zhao & Cao, 2022). This genus is primarily distributed in the Tibetan Plateau and its adjacent areas, it has been included in the national pharmacopeias of 19 countries. The Qinghai‐Tibet Plateau has the original community of rhubarb, the evolutionary community of rhubarb, and some transitional communities, which should be the origin and differentiation center of rhubarb (Wang, 2011). Therefore, we chose rhubarb for our research and analyzed the relationship between niche breadth and distribution range, as well as the correlation between niche characteristics and phylogeny, to provide clues for the evolutionary trend of ecological specialization.

In this study, we collected records of eight species of rhubarb that were distributed in the Qinghai‐Tibet Plateau. Different methods and metrics have been used in niche models to characterize the niche breadth of rhubarbs. Their phylogenetic relationships were reanalyzed to obtain genetic distances, and the distribution of suitable areas for rhubarb in different scenarios in the future was analyzed. The purpose of this study was by to (1) explore the relationship between niche breadth and the current range of suitable areas and fluctuations of suitable areas under different conditions in the future, (2) assess the correlation between niche similarity and phylogeny, and (3) identify key climatic factors and future spatial variation patterns of rhubarb species evolution and formation.

## 2 MATERIALS AND METHODS

### 2.1 Occurrence data and processing

The conditions for our collection of eight species of rhubarb are (1) distributed on the Qinghai‐Tibet Plateau and (2) collect the existing records in the database as of 2022. To collect the occurrence records of *Rheum tanguticum*, *Rheum przewalskyi*, *Rheum moorcroftianum*, *Rheum spiciforme*, *Rheum nobile*, *Rheum officinale*, *Rheum palmate*, and *Rheum pumelo*, data were obtained from the following sources: China Digital Herbarium (https://www.cvh.ac.cn/), CNKI database (http//www.cnki.net/), China Plant Image Library (http://ppbc.iplant.cn/), NSII China National Specimen Resource Platform (http://www.nsii.org.cn/2017/home.php) and field investigation as well. When a record was missing precise geographic coordinates, we used Google Earth (http://ditu.google.cn/) to determine the latitude and longitude based on the geographic location described, and 1105 records were collected. To remove duplicate records and spatially filter, only one point (buffer radius of 20 km) remained within each grid cell after analysis by ENMTools. After removing redundancy, a total of 988 data distribution points were retained. The specific distribution point information is presented in Table 1. The latitude and longitude coordinates of the sample were converted into CSV format.

**TABLE 1:** Geographical range and plot information of eight species of rhubarb on the Qinghai‐Tibet Plateau.

Species	Plot number	Optimized point number	Longitudinal range(°E)	Latitudinal range(°N)
*R. palmatum*	345	268	88.0–117.0	28.8–40.6
*R. tanguticum*	320	306	91.1–112.8	29.0–38.3
*R. pumilum*	213	199	88.7–104.2	28.1–39.5
*R. spiciforme*	57	55	75.0–101.5	28–38.9
*R. moorcroftianum*	48	47	76.4–101.4	28.2–39.5
*R. nobile*	45	41	87.6–102.0	27.3–29.9
*R. przewalskyi*	40	40	92.9–102.9	31.4–39.9
*R. officinale*	37	32	99.0–111.9	25.8–33.9

### 2.2 Sequence collection and analysis

We searched the GenBank database (https://www.ncbi.nlm.nih.gov/genbank/) from January 2000–2021 for rhubarb chloroplast DNA sequences: NADH dehydrogenase subunit F (ndhF), partial cds; voucher trnS‐trnG intergenic spacer, complete sequence; tRNA‐Lys (trnK) gene, partial sequence; maturase K (matK) gene, complete cds; ribulose‐1,5‐bisphosphate carboxylase/oxygenase large subunit (rbcL), partial cds; and rbcL‐accD intergenic spacer, partial sequence (Table S1). Our strategy included (a) sequences that could be linked to all species, and (b) sequences that could be assigned to a set of geographic coordinates.

We used the DNA sequences shared by the eight rhubarb species for alignment. The trnK and matK, trnS‐trnG, and rbcl trees were constructed using maximum likelihood in MEGA 7 (Table S2). To obtain statistical support for the resulting clades, we performed multiple sequence alignments and then used the neighbor‐joining method for tree building; a bootstrap analysis with 1000 replicates was performed. Evolutionary distance is the number of nucleotide substitutions that occur between different sequences. In the phylogenetic tree, pairwise distance was exported to calculate the evolutionary distance, which was used for further correlation analysis.

### 2.3 Acquisition of climate data and environmental variables

The current model uses the CCSM4 climate change model data. For future climate scenarios, we used the CCSM4 climate change modeling data underlying the representative concentration pathways (RCPs): RCP2.6, RCP4.5, and RCP8.5, released by the Intergovernmental Panel on Climate Change (IPCC)‐CMIP5. These RCPs represent the full range of possible values for total radiative forcings of +2.6, +4.5, and + 8.5 W/m^2^, respectively. The two selected future climate datasets (2050 and 2070), were downloaded from the World Climate Database (https://www.sciencebase.gov/catalog/item/) with a spatial resolution of 30 arc seconds. All bioclimatic variables were converted to the ASCII format for MaxEnt analysis. Nineteen bioclimatic and elevation variables were downloaded from the WorldClim Global Climate Database (version 1.4) (http://www.worldclim.org) at a spatial resolution of 30 arcseconds (approximately 1 km). These are the climatic variables that are most widely used in species distribution models. Eight soil variables were downloaded from the National Tibetan Plateau Data Center (https://data.tpdc.ac.cn/zh‐hans/) (Table S3). These variables were collected at a spatial resolution of 30 arcseconds. A base map of China was obtained from the National Basic Geographic Information System (http://nfgis.nsdi.gov.cn).

There is a severe multicollinearity problem among the bioclimatic variables. To screen out the model variables with strong predictive ability and eliminate the multi‐linear relationship between variables, we used Chinese territory‐related data to establish Pearson correlation coefficients for 28 environmental variables using EMTools software. Then, we excluded environmental variables with a correlation >0.8 among environmental variables, and excluded environmental variables with a small contribution rate. The retained environmental variables were used for further model prediction.

### 2.4 Calculation niche breadth and niche overlap

ENMTools v1.4 software was used to calculate the niche breadth of each species, the average value of Levins B1 (inverse concentration) and B2 (uncertainty) values in the habitat suitability map of each species. The values of Levins B1 and B2 range from 0 to 1, with a value close to 1, thus indicating a wider niche breadth and a value close to 0 indicating a narrower niche breadth. ENMTools software was further used to assess niche overlap and range overlap using two indices, Schoener's D and Hellinger's I. The value of niche overlap and range overlap ranges from 0 to 1, with values close to 1 indicating a high degree of niche overlap and range overlap and values close to 0 indicating a low degree of niche overlap and range overlap (Jiang et al., 2021).

$$\text{Overlapping degree}=\frac{A_{\text{overlap}}+B_{\text{overlap}}}{A_{\text{total}}+B_{\text{total}}}$$

### 2.5 Model construction and selection

Using the MaxEnt model to predict the potential distribution of rhubarb. We randomly selected 75% of the occurrence records as training data for model construction, and the remaining 25% as test data for model evaluation The “create response curve” option was selected and the model was repeated 5 times with a maximum number of iterations of 5000. A jackknife was used to evaluate variable contributions. We used a “10 percentile training presence logistic threshold” to define habitats suitable and unsuitable for species distributions. The other parameters used the default settings of MaxEnt. The AUC of an ROC curve is a threshold‐independent measure of model accuracy that is within the threshold, and lists both correct and incorrect predictions. The area under the curve (AUC) was calculated as the area enclosed by the ROC curve, and the value was between 0 and 1. Model performance was classified as failed (0.5~0.6), poor (0.6~0.7), average (0.7~0.8), good (0.8~0.9), or excellent (0.9~1.0). An AUC value closer to 1 indicates better model performance. The AUC values obtained by MaxEnt were >0.9, the standard deviation was <0.02, respectively, the model simulation was found to be “excellent”. Minimum training presence was used to divide the fitness index into four levels: unsuitable, low suitability, medium suitability, and high suitability (Table S4).

## 3 RESULTS

### 3.1 Niche breadth and current and future patterns of change

**FIGURE 1:**
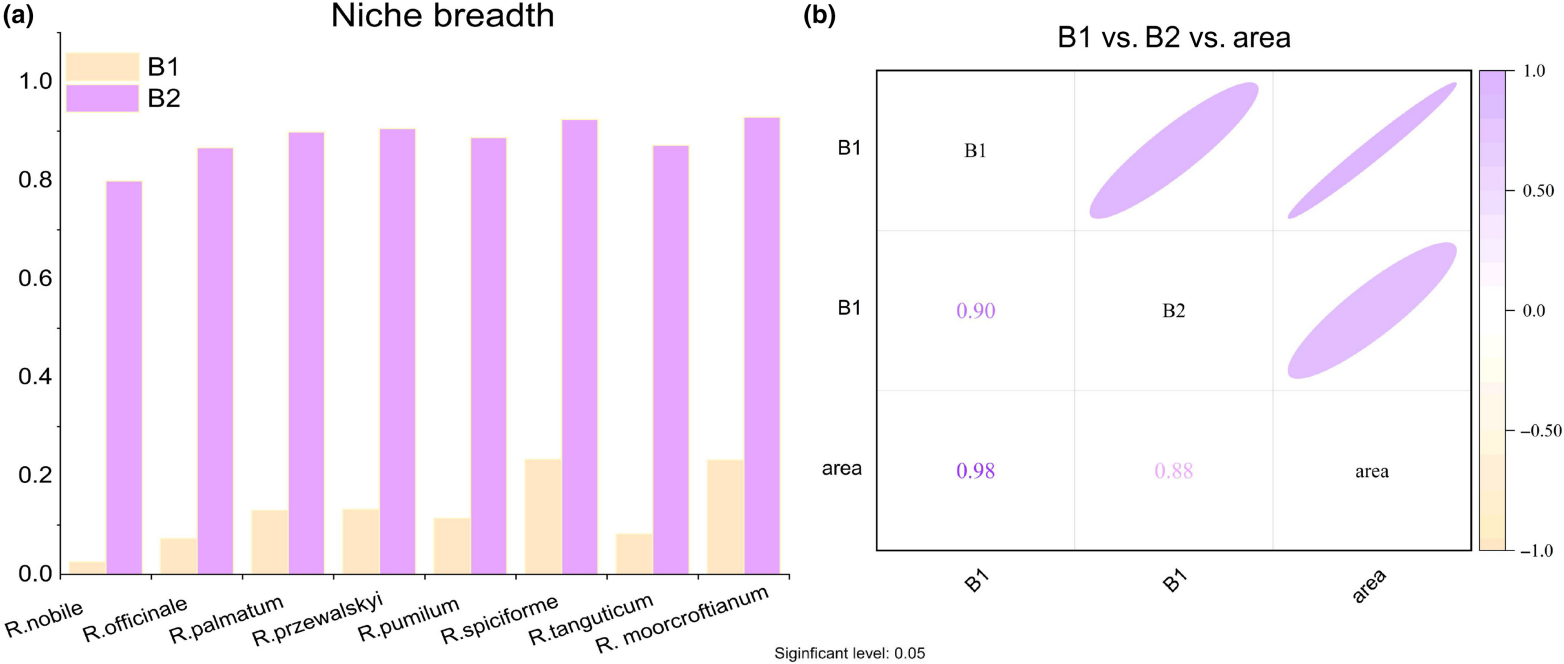
(a) Niche breadth of eight species of rhubarb (B1 and B2). (b) Correlation analysis between niche breadth and current suitable area.

**FIGURE 2:**
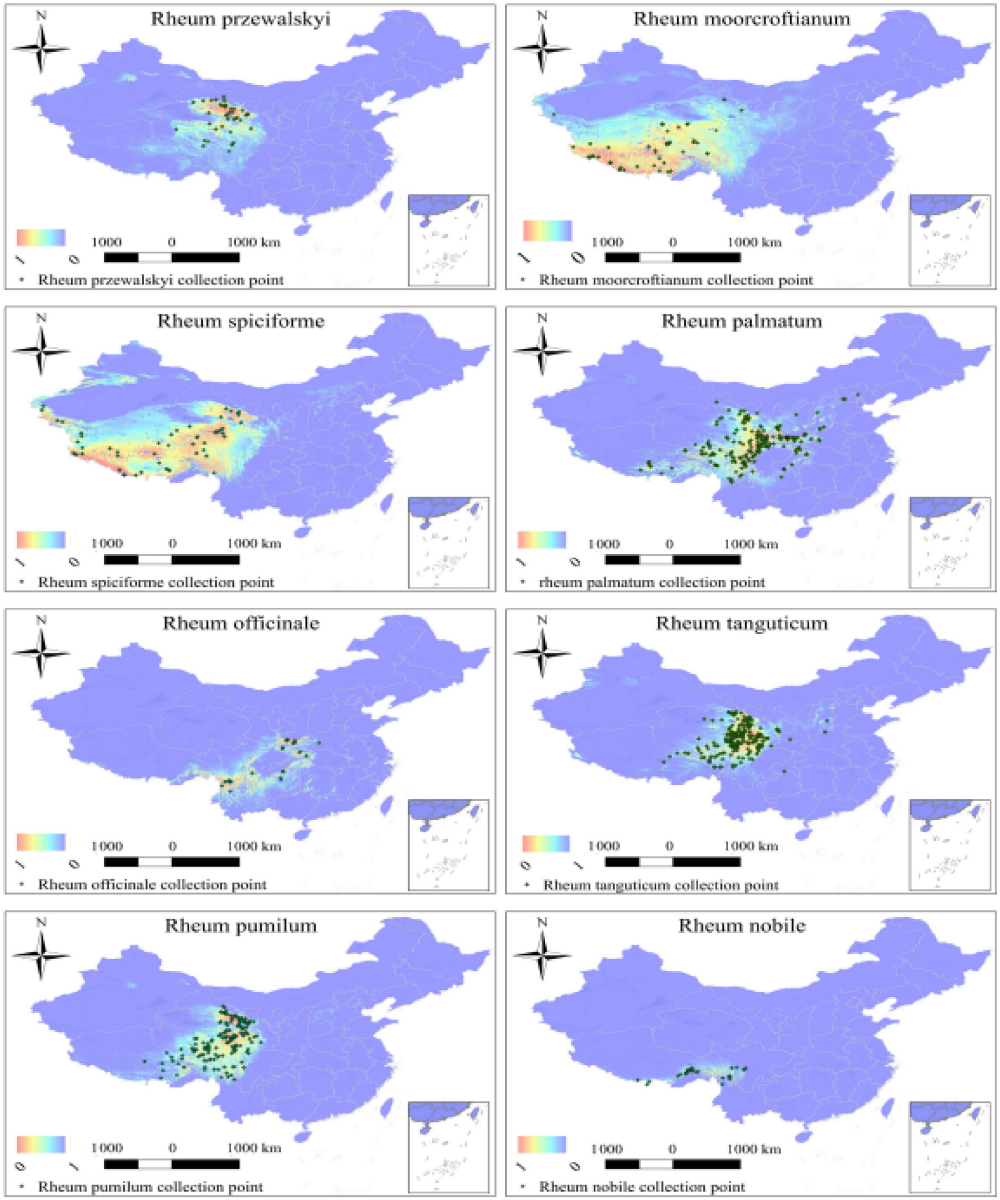
Distribution map of suitable habitats of eight species of rhubarb.

Based on our analysis of the relationship between ecological niche and species distribution, our findings reveal that: *Rheum moorcroftianum* and *Rheum spiciforme* had the widest niches (B1 and B2 were the highest, 0.23 and 0.92, respectively), followed by *Rheum przewalskyi*, *Rheum palmatum*, *Rheum pumilum*, *Rheum tanguticum*, *Rheum officinale*, *and Rheum nobile* (B1 and B2 were 0.02, 0.798, respectively). Specific data are presented in Figure 1 and Table S5. We used ArcGIS software to count the number of eight types of rhubarb to calculate the size of the geographic range (Figure 2), taking 50 × 10^4^ km^2^ as the division interval. The results showed that *R. spiciforme*, *R. moorcroftianu* and *R. palmatum* have wider areas. The suitable areas for *R. przewalskyi* and *R. pumilum* were moderate. *R. tanguticum*, *R. officinale*, and *R. nobile* were smaller (Table S6).

To test whether the fluctuation of the suitable area of rhubarb in the future is related to the niche breadth, we performed a correlation analysis based on the niche breadth (B1, B2) and the changes in the area of the six highly suitable areas in 2050 and 2070 (Table 2). Except for RCP4.5 in 2050, the floating range of suitable areas in the other five future scenarios is negatively correlated with niche breadth. However, in 2050, the correlations under the three conditions were not significant, and in 2070, all three conditions were highly correlated (*p* < .01) (Figure 1).

**TABLE 2:** The relationship between the change ratio of the suitable area of rhubarb and the niche breadth under six future scenarios.

		50RCP2.6	70RCP2.6	50RCP4.5	70RCP4.5	50RCP8.5	70RCP8.5
B1	Pearson correlation	−0.092	−0.58	0.6	−0.56	>−0.01	−0.58
	Sig	0.83	0.13	0.11	0.15	0.98	0.13
B2	Pearson correlation	>−0.01	−0.85	0.46	−0.84	−0.12	−0.83
	Sig	0.97	<0.01	0.25	<0.01	0.77	<0.01

### 3.2 The relationship between niche overlap and phylogeny

**FIGURE 3:**
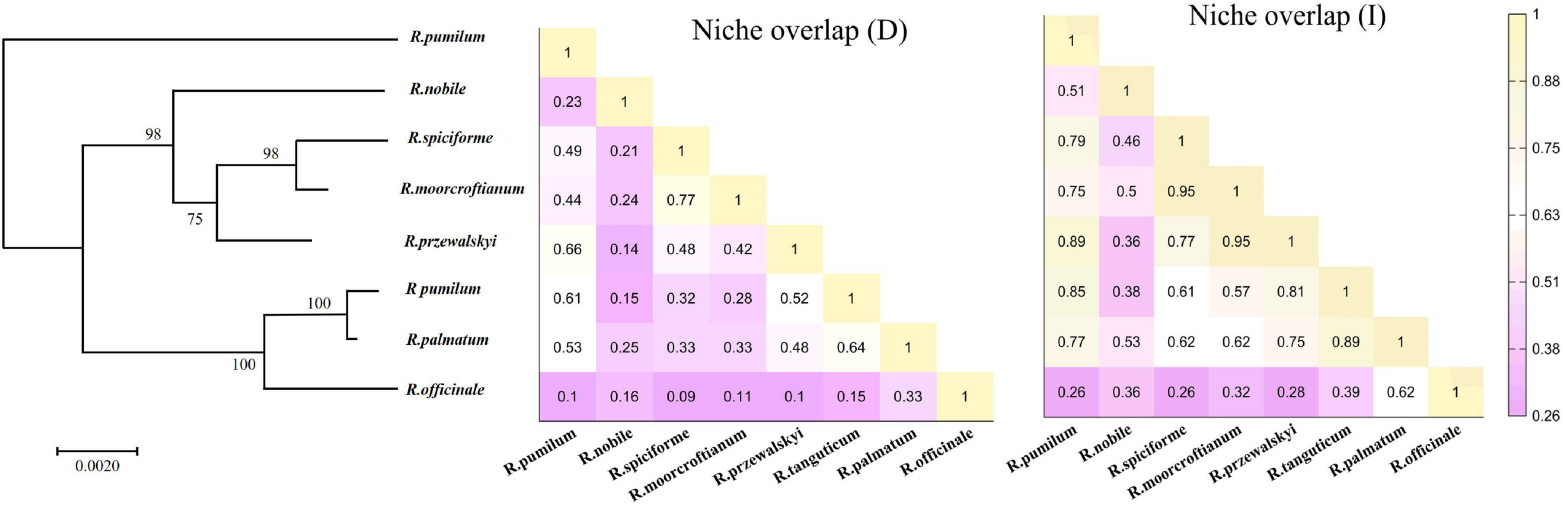
Reconstructed phylogenetic tree and niche overlap values of rhubarb (D and I).

According to the phylogenetic relationship among the eight rhubarb species, the following results can be obtained: *Rheum spiciforme*, *Rheum moorcroftianu* and *Rheum przewalskyi* are in the same clade and were sister species to each other. *Rheum palmatum*, *Rheum officinale*, and *Rheum tanguticum* are sister species (Figure 3). This is the same result as the Losina‐Losinskaya (1936) study.

**FIGURE 4:**
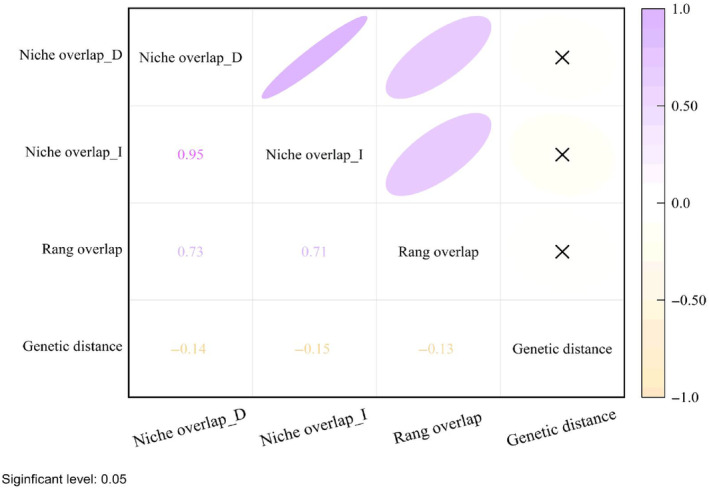
Correlation plot between niche overlap and range overlap and genetic distance.

We generated three scenarios for the niche‐phylogenetic distance relationship: one for niche overlap of the two types (I and D) for each similarity measure and one for geospatial extent overlap. According to the assessment of niche overlap, the highest overlap was observed between *R. moorcroftianu* and *R. spiciforme* (I: 0.95, D: 0.77), followed by *R. przewalskyi* and *R. pumilum* with an ecosystem overlap (I: 0.89, D: 0.66) (Figure 3). In terms of range overlap, *R. spiciforme* demonstrated a significant degree of overlap with most species. The highest overlap was observed with *R. moorcroftianu* at 0.86, followed by *R. nobile* and *R. pumilum* with a range overlap of 0.78. On the other hand, *R. officinale* and several other species exhibited a relatively low range overlap. Specifically, *R. przewalskyi* had a range overlap value of 0, while *R. pumilum* and *R. tanguticum* had very low range overlaps of 0.005 and 0.02, respectively. (Table S7). The mean Pearson correlation coefficient between genetic distance, range overlap, and niche overlap was −0.14, and the mean correlation coefficient was −0.6. There was no significant correlation between niche overlap and the genetic distance between lineages (*p* > .05) (Table 3 and Figure 4).

**TABLE 3:** Correlations between niche overlap and range overlap and genetic distance.

	Range overlap vs. Dist	I_overlap vs. Dist	D_overlap vs. Dist
Pearson correlation	−0.132	−0.156	−0.14
Sig	0.502	0.429	0.476
Correlation coefficient	−0.046	−0.043	−0.026
Sig	0.818	0.639	0.897

	Range overlap vs. D_overlap	I_overlap vs. D_overlap	Range overlap vs. I_overlap
Pearson correlation	0.735**	0.954**	0.711**
Sig	0	0	0
Correlation coefficient	0.720**	0.963**	0.673**
Sig	0	0	0

Note: **Significance at *p* < .01.

### 3.3 Important environmental factors of eight species of rhubarb

**FIGURE 5:**
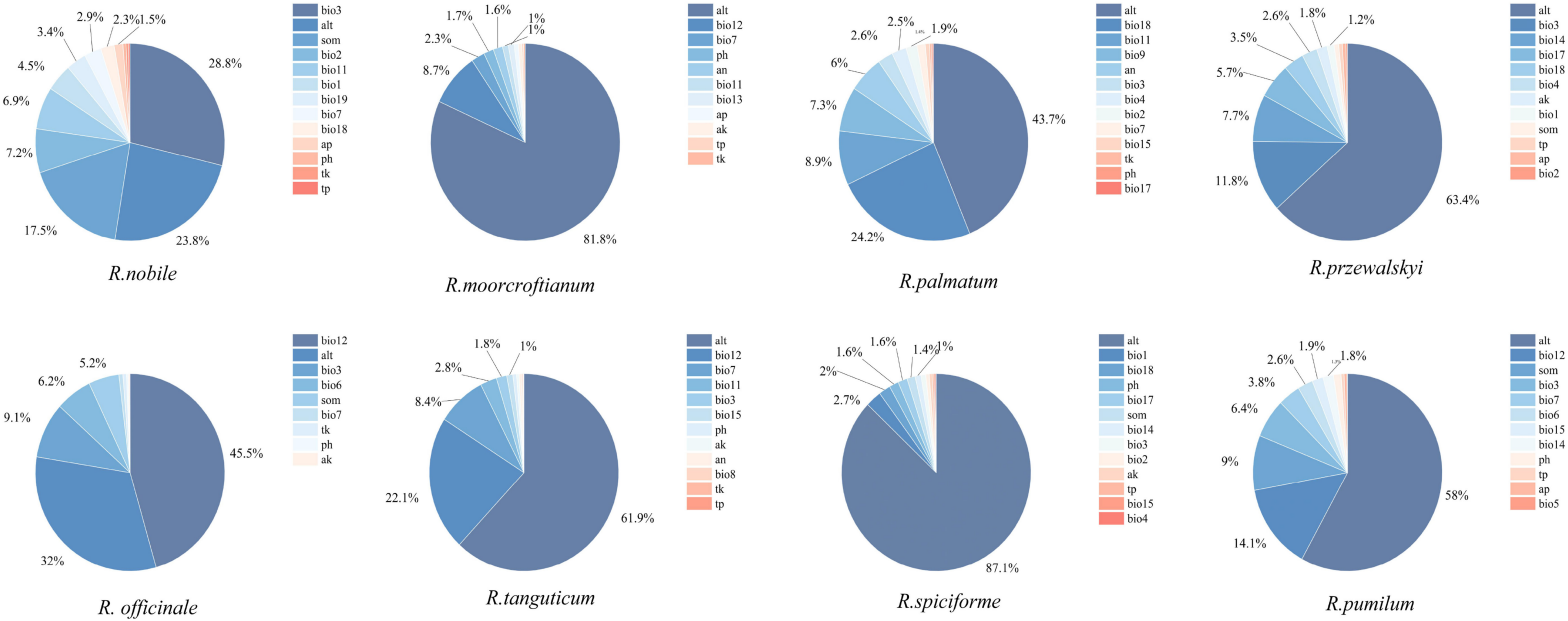
Contribution of environmental factors to the growth and development of eight species of rhubarb.

The environmental factors of the eight rhubarb species surveyed had different contributions (Figure 5), and elevation (alt) was the most important environmental factor affecting the distribution of rhubarb (except for *R. nobile* and *R. officinale*). Climate (temperature and precipitation) was the second factor affecting the growth distribution of rhubarb. The order of the proportion of climate in the contribution of the total factors affecting the growth of rhubarb is as follows: *R. officinale* (61.5%), *R. nobile* (55.9%), *R. palmatum* (49.4%), *R. tanguticum* (39.3%), *R. przewalskyi* (33.7%), *R. pumilum* (23%), *R. moorcroftianum* (13%), and *R. spiciforme* (9.2%). Soil was the least significant contributing factor. Among the eight soil factors, soil organic matter (som) contributed relatively more compared to other soil factors, particularly for *R. nobile* (17.4%), *R. pumilum* (9%), and *R. officinale* (5.2%).

We further analyzed four factors with larger contributions: elevation (alt), annual mean temperature (bio1), isotherm (bio3), and annual precipitation (bio12). Based on kinship analysis of sister species, the most suitable interval of environmental factors was determined. The results showed that some closely related rhubarb species have the same suitable range for certain environmental factors. For example, *R. spiciforme*, *R. moorcroftianum*, and *R. przewalskyi* of *sect. Spiciformia* are all suitable for growth in environments with lower temperature and less precipitation, but the suitable elevation of the three kinds of rhubarb are different, which are 6100.9 m, 5204.8 m, and 3583.9 m, respectively. Both *R. palmatum* and *R. tanguticum* of *sect. Palmata* are suitable for growing at an elevation of 4000 m, but their suitable temperatures and precipitation are different. The suitable annual average temperature of *R. palmatum* is 3.1°C, and the annual precipitation is 857.5 mm; the suitable annual temperature of *R. tanguticum* is 1.1°C, and the annual precipitation is 545.3 mm; *R. palmatum* and *R. officinale* have similar growth environments, but different elevation. *R. officinale* is suitable for growing at 2986 m. corresponded to the specific suitable ranges for each rhubarb (Table S8).

### 3.4 Future rhubarb distribution habitat distribution model

**FIGURE 6:**
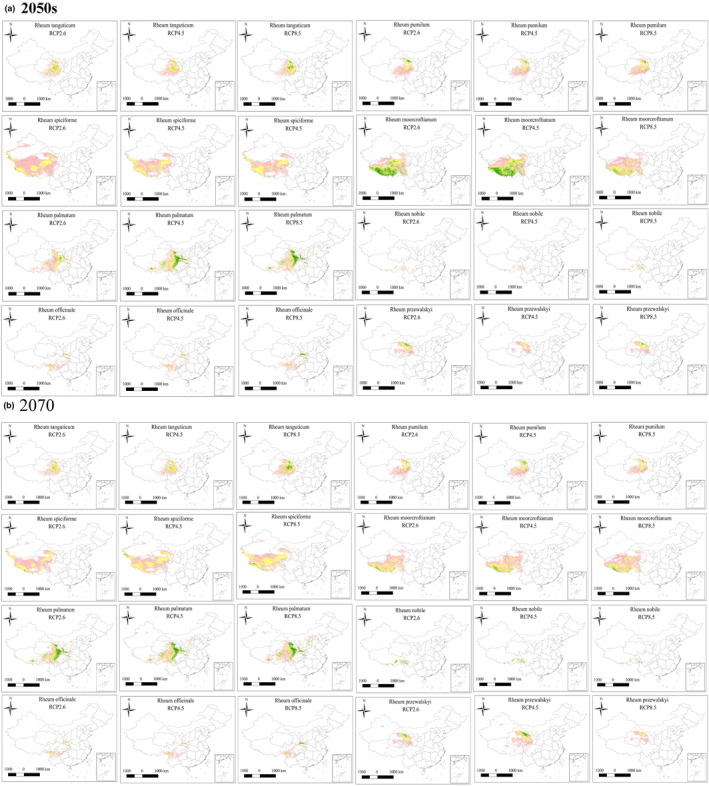
(a) Prediction of the suitable range of the eight species of rhubarb in 2050. (b) Prediction of the suitable range of the eight species of rhubarb in 2070. RCP, representative concentration pathway.

In the future, the total suitable area of *Rheum spiciforme* will decrease in any scenario, and the higher the concentration of greenhouse gases, the smaller the suitable area. The suitable area of *Rheum przewalsky* is similar under medium and low levels of greenhouse gas emissions, and the suitable area was the lowest under the high emission scenario, and the area decreased with increasing years. In addition to the increase in the area of *Rheum nobile* in 2050 under the RCP2.6 scenario, the area of the other five scenarios will decrease, but the area of highly suitable areas will generally increase, and the areas of medium and low suitable areas will decrease. In the future, except for the reduction of the suitable area of *Rheum tanguticum* under the RCP2.6 scenario, the area of the other five scenarios will increase, but the area of the highly suitable area will decrease, and the area of the medium‐low suitable area will increase. The area of *Rheum pumilum* will decrease under any scenario in the future, and the area of suitable habitat will decrease with an increase in greenhouse gas concentrations and an increase in years. The suitable area of *Rheum officinale* will decrease in the future, but when greenhouse gas emissions are the largest, the suitable area will be relatively high, and the highly suitable area will also increase relative to the current area. In addition to scenario RCP2.6, the suitable area was smaller than the current area, but the other five conditions increased, the suitable area increased with increased greenhouse gas concentration, and the area of the suitable area decreased with increasing years. In the future, the total suitable area of *Rheum palmatum* will decrease in any scenario, showing that the higher the concentration of greenhouse gases, the smaller the suitable area (Figure 6). The areas and proportions of the future suitable areas are listed in Table S6.

## 4 DISCUSSION

In this study, we found a significant positive correlation between habitat size and niche breadth of eight species of rhubarb distributed on the Qinghai‐Tibet Plateau, which is consistent with the results reported by many previous studies (Brown, 1984; Cai et al., 2021; Kirkpatrick & Barton, 1997). Species with large niches are located in wider habitats and tend to have larger distribution ranges and overall population sizes (Kirkpatrick & Barton, 1997; Williams et al., 2006), which can provide a higher chance of speciation events, both quantitatively and qualitatively (Harnik & Payne, 2012). The more extensive the habitat, the greater the likelihood of local adaptation processes; therefore, the positive correlation between range size and niche breadth is not surprising (Calixto‐Rojas et al., 2021; Gaston, 1991; Slatyer et al., 2013). With the growth of the time scale, the superiority of the niche breadth can be more reflected (Pearman et al., 2008), species with a narrow niche are thought to be more vulnerable than generalists to current and future climate change (Devictor et al., 2010; Saupe et al., 2015), and species with wider niches have a stronger ability to cope with environmental changes (Colles et al., 2009), which may be the reason why the future suitable range of species with wider ecological niches fluctuates less. In the future, except for *R. tanguticum* and *R. palmatum*, the suitable areas for the other six species of rhubarb will decrease, which is characterized by a decrease in suitable areas with an increase in the concentration of greenhouse gas emissions and the passage of time. Alpine ecosystems are highly sensitive to climate change, and biotic communities are often restricted by low temperatures. (Evans et al., 2009; He et al., 2019), The adaptation of species to these changes may be slower than the pace of climate change itself, which can result in a reduction in their distribution range and/or local extinction (Pauli et al., 2010; Rumpf et al., 2018). Temperature is an important factor affecting distribution (Wang et al., 2020), and species with narrow ecological niches face serious threats as temperatures increase with global change (Lenoir et al., 2008).

Niche conservation is defined as the tendency of genetically similar species to maintain ecological similarity during evolution (Losos, 2010; Wiens & Graham, 2005). In general, closely related plants have evolved similar adaptation mechanisms to living environments (Losos, 2010; Rundell & Price, 2009), combining climate and topography, including precipitation and temperature, with sister species sharing important environmental factors and corresponding similar optimal values (Peterson et al., 1999; Rundell & Price, 2009). In this study, for example *R. moorcroftianum* and *R. przewalskyi*, *R. moorcroftianum* and *R. spiciforme*, and *R. tanguticum* and *R. palmatum* were sister species with relatively close kinship, and their niche overlap was high. However, despite similarities in niche breadth and impact factors of the closely related rhubarb, there are also species with highly overlapping niches that are neither sister species nor in the same group. Such as *R. tanguticum* and *R. pumilum*, *R. pumilum* and *R. przewalskyi*. Furthermore, the niche overlap of the eight rhubarb species in this study had a low correlation with the genetic distance, range overlap, and genetic distance (*p* > .05), and the same phenomenon was also found in the range overlap (*p* > .05). There are also cases with similar functional niches but distributed in different geographical areas, and the optimal range of environmental factors is similar but not sister species, such as *R. tanguticum* and *R. nobile*, which are considered to be ecologically equivalent organisms. Thus, niche similarity among rhubarb species was not significantly related to the phylogenetic structure of this population. This may be because of the principle of competitive exclusion, where niche overlap between closely related species creates habitat competition to a certain extent in stable environments (Anacker & Strauss, 2014; Jordan, 1905). Considering that two species occupy the same ecological niche, two species with overlapping niches cannot coexist for long periods of time unless space and resources are abundant (Noden, 1986; Penny, 2004). The niche conservation theory argues that if a group of organisms reflects differentiation rather than conservation, ecological dimensions should be considered to have a greater impact on the evolution of those organisms (Calixto‐Rojas et al., 2021; Knouft et al., 2006; Peterson et al., 1999). Our results show that the ecological dimension has a greater impact on the speciation of eight rhubarb species on the Qinghai‐Tibet Plateau than on geographic latitude.

Alpine plants are the result of plants adapting to extreme living environments after mountain uplift (Su, Farnsworth, et al., 2019). Wang (2011) suggested that the Qinghai‐Tibet Plateau was the birthplace of rhubarb, and it was difficult for rhubarb to survive at low elevation. Similarly, in this experimental study, it was found that the most important factor affecting the evolution and formation of rhubarb species was elevation. Elevation generally interacts with temperature and light, and has significant effects on the accumulation of metabolites and nutrients in rhubarb. These eight species of rhubarb are suitable for growing at an elevation of more than 2800 m. This is because rhubarbs are prone to bolting when grown at elevation below 1500 m (Huang & Jiang, 2019). Bolting flowers early and lignifying the root system has a significant impact on growth and development. Climate is the second factor affecting the growth of rhubarb plants. Because the leaves of rhubarb are large and thin and water evaporates quickly, it is not conducive to growth and development in an environment that is too dry and windy (Mohtashami et al., 2021). Humid climate and soil are also extremely unfavorable for the growth and development of rhubarbs, and the roots are easily rotten. Therefore, rhubarb is suitable for growing in a slightly dry and humid climate and slightly shaded environment (Wang, 2011). Furthermore, our research has revealed that the Hengduan Mountains region serves as a habitat for all eight species of rheum, which includes the widely distributed *Rheum spiciforme* as well as the ecologically limited *Rheum nobile*, despite their contrasting ecological requirements. The Hengduan Mountains are located on the southeastern edge of the Qinghai‐Tibet Plateau, where the Jinsha, Lancang, and Nujiang Rivers run in parallel (Boufford, 2014), forming a complex landform with large height differences and a vertical vegetation belt (Eldrett et al., 2009; Su, Spicer, et al., 2019). The alpine biodiversity of the Hengduan Mountains is a hotspot among biodiversity hotspots (Boufford, 2014; Madriñán et al., 2013; Xin‐Hui et al., 2014), and is considered to be the cradle of the origin and differentiation of alpine species (Ding et al., 2020), as well as the main source of alpine diversity in the Himalayas and Qinghai‐Tibet (Madriñán et al., 2013; Rundell et al., 2004; Tu et al., 2010; Xin‐Hui et al., 2014). Therefore, we recommend designating the Hengduan Mountains as a key protected area to protect *Rheum* species. Protection measures could include restricting human activities, strengthening monitoring and management, promoting the implementation of environmental protection regulations, and so on. At the same time, further scientific research should be conducted to increase our understanding of *Rheum* growing in the area, in order to develop more effective conservation strategies.

## 5 CONCLUSIONS

This study analyzed the relationship between phylogeny and niche using eight species of rhubarb distributed on the Qinghai‐Tibet Plateau and predicted the correlation between the range of the current suitable area, the variation range of the future suitable area, and niche breadth. The results show that species with similar niches are not necessarily sister species, and there is no significant relationship between niche overlap and phylogeny. We infer that ecological dimension has a greater impact on speciation of the eight rhubarb species than geographic latitude. Niche breadth is positively correlated with current suitable areas and negatively correlated with future suitable area fluctuations. We predict that species with small niches are at greater risk of habitat loss than those with large niches.

## AUTHOR CONTRIBUTIONS

**Kaiyang Chen:** Conceptualization (lead); formal analysis (lead); software (lead); visualization (lead); writing – original draft (lead). **Bo Wang:** Data curation (lead); formal analysis (supporting); investigation (supporting); resources (lead); software (supporting); writing – review and editing (lead). **Chen Chen:** Methodology (supporting); project administration (supporting); supervision (supporting); validation (supporting). **Guoying Zhou:** Conceptualization (lead); funding acquisition (lead); supervision (supporting); writing – review and editing (supporting).

## CONFLICT OF INTEREST STATEMENT

The authors declare that they have no known competing financial, interests or personal relationships that could have appeared to influence the work reported in this paper.

## Supporting information


Appendix S1.

## Data Availability

Data distribution points of eight rhubarb species may be available from the authors on reasonable request.
